# Chemical and Biological Evaluation of the Oil and Seedcake from Seeds of a Greek Cardoon Cultivar as Potential Functional Vegetable Oil. Comparison with Sesame, Flaxseed and Extra Virgin Olive Oils

**DOI:** 10.3390/foods10112665

**Published:** 2021-11-02

**Authors:** Elisavet-Foteini Varvouni, Konstantia Graikou, Olga Gortzi, Antigoni Cheilari, Nektarios Aligiannis, Ioanna Chinou

**Affiliations:** 1 Laboratory of Pharmacognosy and Chemistry of Natural Products, Department of Pharmacy, National and Kapodistrian University of Athens, 15771 Athens, Greece; elisavet_v@hotmail.gr (E.-F.V.); kgraikou@pharm.uoa.gr (K.G.); cheilarianti@gmail.com (A.C.); aligiannis@pharm.uoa.gr (N.A.); 2 Department of Agriculture Crop Production and Rural Environment, University of Thessaly, 38221 Volos, Greece; ogortzi@gmail.com

**Keywords:** *Cynara cardunculus*, seed oil, seedcake, linoleic acid, total phenolic content, total lignan content, nutritional value, antioxidant activity, enzyme inhibitory activity

## Abstract

*Cynara cardunculus* L. is a plant of the Mediterranean basin, known since antiquity as a food and for its therapeutic properties. The needs of the 21st century for the utilization of agricultural waste has led to the study of the seed oil of a Greek cultivar of *Cynara* *cardunculus* (GCCC) as potential nutritional oil, as large amounts of cardoon seeds are discarded. The sterol and fatty acid profile of cold-pressed seed oil was examined by gas chromatography-mass spectrometry GC-MS and compared with that of solvent extraction. Total phenolic content was determined and compared with well-known and widely appreciated edible vegetable oils; while, additionally, the total lignan content and nutritional value of cold-pressed oil revealed it as a potential dietary candidate. Furthermore, the seedcake (residue of cold-pressed oil extraction) has been studied exerting it as a good source of phenolics. Both GCCC oil and seedcake were tested for their antioxidant and enzyme inhibitory activities exhibiting higher activity compared to the sesame, flaxseed and extra virgin olive oils. According to the results, *Cynara* seed oil was shown to be a rich source of ω-6/-9 fatty acids and phenolics, highlighting, indicating that it could be a promising health-promoting vegetable oil, while the seedcake was revealed as a rich source of bioactive compounds.

## 1. Introduction

The needs of the 21st century for food and energy have led to the finding of new alternative strategies of the utilization of agricultural and food waste by-products [1]. Agronomic and food wastes can be proved as potential pools of bioactive metabolites, beneficial for human nutrition and health, such as essential fatty acids, phenolics, dietary nutrients etc. [2,3]. Thus, the exploitation of such wastes with value added potential is a very important factor for a sustainable circular agricultural economy [4].

*Cynaracardunculus* (Asteraceae) includes artichoke and cardoon, where cardoon includes two varieties, cultivated and wild [5]. It is a perennial plant species originating from the Mediterranean basin and gaining attention as a multipurpose crop [6]. *C. cardunculus* has been used for culinary and medicinal purposes since antiquity due to its beneficial health effects. In several studies, its anti-inflammatory, antidiabetic, cardiotonic and cholesterol-lowering effects have been highlighted, while it is also referenced as an important source of bioactive compounds and nutrients [6,7]. Several studies have focused on the cultivation of *C. cardunculus* plants for industrial purposes, particularly as a bioenergy crop, due to its low cultivation requirements in terms of water and nutrients, and its high-biomass yield and calorific value [5,8]. According to the literature, cardoon seeds are considered a rich source of minerals, macroelements, lignans, sterols, fatty and phenolic acids, but also its seed oil is considered a rich source of unsaturated fatty acids [5,8,9,10,11,12,13], of which ω-6 linoleic acid is most abundant (ranges between 56.7–64.8%), followed by monounsaturated oleic acid (21.11–28.4%) and saturated palmitic and stearic acids (9.37–11.1 and 2.78–3.7%, respectively), indicating that this oil could be suitable for consumption [5,14].

In the last decades, plant-derived oils have been generally approved as a ground-breaking area of the market, as they are not only used in the food industry, as a cooking oil and/or as a substitute for animal-derived products and in cosmetics, in soaps and related items, but also as sources of bioactive supplements supporting the maintenance of human health [15]. In addition, according to a market-research company (IMARC) the global vegetable-oil market grew at a compound annual growth rate (CAGR) of 3.6% over 2014–2019, with consumption volume reaching 207.5 million tons in 2019. This increasing demand of the market can be attributed to the fact that dietary habits have been significantly altered in the recent years. 

Among the edible vegetable oils, olive oil (*Olea europaea* L., Oleaceae) is the primary source of fats in the Mediterranean diet. Its consumption is associated with lower cardiovascular risk, cancer and neurodegeneration, with much evidence that it positively affects human health, due to its nutrients [16]. Sesame seed (*Sesamum indicum* L., Pedaliaceae) is considered a major oil source, with a good taste, high nutritional value and therapeutic properties [17,18]. Fatty acids, such as linoleic acid, combined with other constituents in sesame oil such as phytosterols, tocopherols and lignans, have been analyzed for their biofunctions in human diets [19]. Flaxseed oil (linseed oil) is another seed-derived oil, obtained from *Linum usitatissimum* L. (Linaceae) and it has also been characterized as a “functional food” due to its nutritional value and its significant concentrations of essential fatty acids [18].

The seeds of GCCC, in a previous study, exhibited very promising outcomes [20], as the seeds have shown remarkably high nutritional value, high percentage of proteins and dietary fibers (19.3 and 24.1%, respectively) and a rich bioactive metabolites profile consisting mostly of fatty acids, lignans and other phenolics. 

Therefore, the aim of the present study was the chemical analysis of the oil derived from these seeds and its comparison with well-known vegetable oils, such as sesame, flaxseed and extra virgin olive oils, as well as the analysis of the seedcake, which is the residue of cold-pressed seed-oil extraction, considered a by-product. The GCCC oil was obtained through two assisted methods: by solvent extraction (SE) and by the green eco-friendly mechanical cold-press extraction (CPE), wherein the latter is a popular method for oil production, having several advantages. CPE is a low-cost technique resulting to an unrefined, ready-for-sale oil, free from chemical contaminants. The oil obtained from CPE usually shows lower oil yield than oil obtained from SE, but it is preferable and suitable for use in the food, cosmetic and/or pharmaceutical industries [21]. Furthermore, the sterol and fatty acid profiles, total phenolic and lignan contents, peroxide values and nutritional values of the obtained oils were evaluated. Τhe two abovementioned oils, together with their seedcakes and six isolated phenolic compounds (five lignans: arctigenin, trachelogenin, arctiin, tracheloside, cynarinine and the phenolic acid: 3,5-dicaffeoylquinic acid) were, in vitro, assayed for their free radical-scavenging activity and enzyme inhibitory activity against acetyl-cholinesterase (AChE). To our knowledge, GCCC oil has never been studied before, while the lignan-content method has never been evaluated before, among all studied *Cynara* species. 

## 2. Materials and Methods

### 2.1. Chemicals and Reagents

All the chemicals and reagents were purchased from: Merck (Darmstadt, Germany) (ethanol absolute, Folin-Ciocalteu reagent, gallic acid, sodium carbonate (Na_2_CO_3_)), Sigma-Aldrich (St. Louis, MO, USA) (sulfuric acid 95–98% (H_2_SO_4_), potassium peroxodisulfate, acetylthiocholine iodide (ATCI), acetylcholinesterase (AChE), 5,5′-dithio-bis(2-nitrobenzoic acid) (DTNB), galanthamine hydrobromide), Sigma (St. Louis, MO, USA) 2,2′-Azino-bis(3-ethylbenzothiazoline-6-sulfonic acid) diammonium salt (ABTS^•+^)), Carlo Erba Reagents (Val-de-Reuil, France) (hexane, petroleum ether, dimethyl sulfoxide (DMSO)), Lab-Scan (Dublin, Ireland) (chloroform), Panreac (Barcelona, Spain) (sodium sulfate (Na_2_SO_4_) anhydrous), Fisher Scientific (Loughborough, UK) (methanol high-performance liquid chromatography (HPLC)-grade, hydrochloric acid 37% (HCl)), Glentham Life Sciences (Corsham, UK) (2,2-diphenyl-1-picrylhydrazyl (DPPH^•^)), Acros Organics (New Jersey, USA) (trolox) and Lachner (Neratovice, Czechia) (sodium hydroxide micropearls (NaOH)), the fatty acids methyl ester (FAME) reference standard mixture 37 (standard 47885) was purchased from Sigma-Aldrich (St. Louis, MO, USA). Sesame oil and linseed oil were supplied by Sigma-Aldrich (St. Louis, MO, USA, tested quality according to Ph. Eur.), while the olive oil (variety Kalamon) was obtained from a Greek producer (Sparti, Peloponnese, Greece).

### 2.2. Plant Material and Seed-Oil Extraction and the Isolation of Their Compounds

The oil was obtained from seeds of an established GCCC. Seeds were separated and collected from the mature heads from the experimental fields of the University of Thessaly Greece, in August 2020 and they were identified botanically by Prof N. Danalatos (Dean of the Department of Agriculture Crop Production and Rural Environment, University of Thessaly, Volos, Greece). A voucher specimen (NoD4) was deposited at the Herbarium of the Lab. of Agronomy and Plant Physiology. The seeds were naturally dried in the shade and stored at 7–10 °C. The moisture content (5.1%) and the fat content (23.7%) of the seeds was determined previously [20].

A portion of them (3.5 kg seeds) were used for CPE, while another portion (50 g seeds) was used for SE. 

For the SE method, the seeds were pulverized and extracted with cyclohexane (3 × 500 mL) for 24 h, each time, at room temperature. The cyclohexane extract was evaporated to dryness on a rotary evaporator under reduced pressure at 40 °C and a yellow oil was obtained (11.15 g) with a yield of 223.0 g oil/kg of seeds. 

For the CPE method, a screw press (GmbH and Co. Kern Kraft KK40), with a seeds capacity of 40 kg/h, was used for pressing the plant material and the oil was obtained without increasing its temperature. Before further analyses, the CPE oil was centrifuged twice (4.000 rpm for 5 min at 25 °C) and the supernatant was kept in a glass bottle (580 g oil) with a yield of 165.7 g oil/kg of seeds. 

The oils obtained from both extraction methods were kept in dark vials and stored in a fridge (4 °C) until analysis.

In addition, 100 g of seeds were subjected to isolation procedure, followed the same chromatographic methods previously described by Graikou et al. [20], to obtain six phenolic compounds, which were identified through NMR spectra (400 MHz) and recorded on a Bruker DRX-400 spectrometer. The isolated compounds were five known lignans: arctigenin (6 mg), trachelogenin (8 mg), arctiin (4 mg), tracheloside (4 mg), cynarinine (3 mg) and the phenolic acid 3,5-dicaffeoylquinic acid (7 mg). All were used as internal standards for this study.

### 2.3. Determination of Sterol and Fatty Acid Composition 

Both studied SE and CPE oils were analyzed for their sterol and fatty acid profiles using standard analytical procedures (involving saponification and methylation) [22] and both fractions were further analysed by GC-MS. 

The analysis was carried out using an Agilent Technologies Gas Chromatograph 7820A (Shanghai, China) linked to Agilent Technologies 5977B mass spectrometer system (Santa Clara, CA, USA) equipped with a 30-m length, 0.25-mm i.d. and 0.5-µm film thickness HP5-MS capillary column. The initial column temperature was 60 °C, which then increased at a rate of 3 °C/min to a maximum temperature of 280 °C, where it remained for 15 min. The total analysis time was 93 min. Helium was used as a carrier gas, at a flow rate of 2.2 mL/min, split ratio of 1:10, injector temperature of 250 °C, and ionization voltage of 70 eV. The identification of components was conducted using mass spectral databases (Wiley275, ADAMS07) and in comparison with existing literature data. 

In parallel, the determination of fatty-acids profiles was performed by gas chromatography C (Varian 450-GC, CA, USA) equipped with a flame ionization detector. The chromatographic column was an Agilent DB-23 Column (length 60 m, id 0.25 mm, film 0.15 μm, Agilent Technologies, Santa Clara, CA, USA) equipped with an Agilent F8 ultimate plus (length 10 m, id 0.25 mm, Agilent Technologies, Santa Clara, CA, USA) precolumn. The initial oven temperature was 50 °C, where it remained for 1 min and then increased, at a rate of 25 °C/min, to 175 °C and, finally, at a rate of 4 °C/min, to a maximum temperature of 230 °C, where it was held for 4 min. Helium was used as a carrier gas, at a flow rate of 1.0 mL/min, an injector temperature of 250 °C, and a detector temperature of 280 °C. Fatty acid identification and quantification was performed by comparing the relative retention times of FAME peaks from samples with those of standards (reference standard mixture 47885).

Moreover, the peroxide index (Commission Regulation (EEC) No. 2568/91 Annex III) was determined as per ISO 3960: 2012 after dissolving the oil sample in an acetic acid- chloroform mixture (3:2, *v/v*) in the presence of a potassium iodine solution. Finally, the mixture was titrated with sodium thiosulfate using starch as an indicator. The results were expressed as meq O_2_ /kg oil.

### 2.4. Seedcake Extraction

A portion of 5 g of seedcake was extracted with ethanol (3 × 50 mL) for 24 h, for each extraction, at room temperature. After filtration, the organic extract was evaporated under reduced pressure to obtain a dry extract for the evaluation of its phenolic content, antioxidant and anti-AChE activities. Additionally, the seedcake was phytochemically screened for the presence of isolated phenolics from the seeds through thin layer chromatography (TLC) and co-spotting method, where the lignans arctiin, tracheloside, cynarinine, arctigenin (traces) and trachelogenin (traces), and one phenolic acid: 3,5-dicaffeoylquinic acid, were detected.

### 2.5. Determination of Total Lignan Content (TLC) of CPE Oil

A chromatographic method, developed by Bhatnagar et al. [23], was used to determine the total lignan content in CPE and SE oils. It is based on the absorption of sample in the ultraviolet spectrum, in relation with the specific absorption value of E^1%^ (specific extinction) of arctigenin, a lignan previously isolated from the seeds of the studied species [20]. The specific extinction value for the lignan arctigenin (E^1%^_1.2 cm_) was calculated according to Bhatnagar et al. [23]. The UV absorbance of the sample was measured at 280 nm (in triplicate) and the results were expressed as mean value± standard deviation. Regarding the preparation of the samples, 0.01 g oils were dissolved in 10 mL of hexane: chloroform solution (7:3, *v/v*).

### 2.6. Determination of Nutritional Value of CPE Oil

The nutritional evaluation of the CPE oil was determined (energy, moisture, proteins, fat, ash and crude fiber content) according to previously described methods [24,25]. Protein content was estimated using the modified Kjeldhal method, while ash and crude fiber content was determined through conventional procedures. Moreover, saturated and unsaturated fatty acids, using GC-FID, were determined. All experiments were performed in triplicate. Details of nutritional estimation parameters have been previously described by Graikou et al. [20].

### 2.7. Determination of Total Phenolic Content (TPC)

The total phenolic content of SE and CPE *Cynara* oils, sesame, flaxseed and olive oils, as well as of seedcake ethanol extract, were determined using the Folin–Ciocalteu method [26]. The total content of phenolic compounds in the tested samples was expressed as gallic acid equivalents (GAE). The experiments were performed in triplicate and the results were expressed as means ± standard deviation (*n* = 3).

### 2.8. Determination of Antioxidant Activity

The studied SE and CPE *Cynara* oils, sesame, flaxseed and olive oils, together with the seedcake and isolated lignans arctigenin, trachelogenin, arctiin, tracheloside, and cynarinine, and the phenolic acid 3,5-dicaffeoylquinic were, in vitro, evaluated for their antioxidant activities using DPPH^•^ and ABTS^•+^ assays. The DPPH^•^ radical scavenging assay was performed according to a previously described method [27]. The ABTS^•+^ radical cation scavenging assay was assayed according to Re et al. [28]. 

The percentage of DPPH^•^ and ABTS^•+^ scavenging was estimated by the following equation:

AA% = {[(A − B) − (C − D)]/(A − B)} × 100,(1)

A: Control (without sample), B: Blank (without sample, without DPPH^•^ /ABTS^•+^), C: Sample, D: Blank sample (without DPPH^•^/ABTS^•+^). All samples were analyzed in triplicate and the results were expressed as means ± standard deviation (*n* = 3).

### 2.9. Determination of Enzyme Inhibitory Properties

The CPE oil and the seedcake were examined for their in-vitro enzyme-inhibitory properties against acetylcholinesterase (AChE).

The AChE inhibitory activity of the samples was evaluated based on the method described in Hasnat et al. [29]. All tested samples were evaluated at 200–50 μg/mL, galanthamine hydrobromide was used as positive control at final concentrations 25 and 1 μΜ and AChE percentage inhibition was calculated by the equation:

inhibition activity (%) = (absorbance of control − absorbance of sample/absorbance of control) × 100,(2)

For all the bioassays the measurements were performed using a TECAN Infinite M1000 PRO multimode reader (Tecan Group, Männedorf, Switzerland) (data obtained with the i-control software, version 1.11).

### 2.10. Statistical Analysis

All samples were evaluated in triplicate and the results were expressed as means ± standard deviation (*n* = 3). Statistical analysis of chemical composition and bioactivity assays was performed with GraphPad Prism 8.3 (San Diego, CA, USA). Data were evaluated by one-way analysis of variance (ANOVA), while the means of values were compared with the Brown–Forsythe and Welch, Dunnett’s and Holm–Sidak methods. The mean comparison for the nutritional value was performed by Duncan’s multiple range test.

## 3. Results and Discussion

### 3.1. Sterol and Fatty Acid Profiles

The analysis of the unsaponified fractions of both the SE and CPE oils showed that sterols, including β-sitosterol, campesterol, stigmasterol, stigmast-7-en-3-ol and taraxasterol, were detected, which is in accordance with the literature regarding *Cynara* spp. [30,31]. The abundant β-sitosterol is a major sterol of the plant kingdom, exhibiting several health benefits, such as cholesterol-lowering effects and a reduced risk of cardiovascular disease. The triterpene, squalene, which is a precursor molecule of sterol biosynthesis, as well as the pentacyclic triterpenes, α- and β-amyrin, were also detected in the studied oils and they have been previously identified in other species of the genus [30]. Squalene is considered to be a molecule with pharmacological added value in cosmetology, due to its emollient, antioxidant and moisturizing properties [32].

In addition, SE and CPE oils showed similarities in their detected fatty acids (Table 1). Both examined oils exhibited a rich profile of unsaturated fatty acids, with linoleic and oleic acid being the major representatives thereof. Moreover, after performing multiple t-tests, using the Holm–Sidak method as well as the false discovery rate (FDR) approach, the two studied oils showed no significant difference in the content of myristic acid (*p* = 0.158), palmitoleic acid (*p* = 0.573), margaric acid (*p* = 0.613) or stearic acids (*p* = 0.170), while a significant difference (*p* < 0.003) was observed for the content of saturated fatty acids (SFA), monounsaturated fatty acids (MUFA), polyunsaturated fatty acids (PUFA) and the ratio PUFA/SFA, as well as for palmitic, linoleic, oleic and arachidic acid. In total, no significant difference was observed between the two studied oils (*p* = 0.670). It is noteworthy that linoleic acid represented approximately 50% of the lipid content of the oils, followed by mono-unsaturated ω-9 oleic acid and saturated palmitic acid. The present results are in accordance with literature data on other cardoon seed oils from different countries, in which linoleic and oleic acids were the predominant acids [5,8,11,12,33]. In addition, stearic acid was detected in lesser amounts, while linolenic and behenic acids were identified only in the SE oil. ω-3 fatty acids were either detected in extremely low amounts or were absent, which is also in accordance with recent studies of *Cynara* seed oil [8]. It was also noticed that the CPE oil contained the highest percentage (24.32%) of saturated fatty acids (SFA), while the highest percentage (58.44%) of polyunsaturated fatty acids (PUFA) was detected in the SE oil.

The high percentage of PUFA provides an incentive to use this oil for health-related reasons, as there is therapeutic evidence of their role in cardiovascular health. Many studies highlight the benefits of PUFA on the improvement of cardiovascular risk or inflammatory problems, such as coronary artery disease, stroke, rheumatoid arthritis and several chronic disorders, such as diabetes and atherosclerosis [34]. Especially, linoleic acid, which belongs to PUFA, is an essential ω-6 nutrient for human diets, involved in health maintenance [34].

Having PUFA/SFA value (2.83 and 1.94 for SE and CPE oils, respectively) is an important advantage factor, as oils with an index higher than 1 are considered edible oils with high nutritional value for humans [5].

Regarding the unsaturated ω-3/-6/-9 fatty acids that characterize the vegetable oils, it was observed that *Cynara* oil contained comparable amounts with the widely consumed sesame and flax-seed oils. Sesame oil is characterized as a vegetable oil of the oleic–linoleic group (approximately 80% of the total unsaturated fatty acids) [35], while flaxseed oil is considered as the richest plant source of linoleic (18–24%) and linolenic (36–50%) acids [36]. The recent literature has reported that the distribution of fatty acids in *Cynara* seed oil was comparable also with other widely consumed oils such as corn, soybean, sunflower and canola oils [5,8].

The peroxide value (PV) of the oil was determined at the beginning of the storage time at 9 meq O_2_/kg oil. Peroxide value (PV) is an important indicator for assessing edible oil quality and the examined oils were of good quality, being in agreement with Codex Alimentarius recommendations [37], which state that the PV for cold-pressed oils should not exceed 15 meq O_2_/kg oil.

### 3.2. Total Lignan Content (TLC)

The total lignan content of CPE oil was 12.53 ± 0.17 g/kg and of SE oil 15.4 ± 0.09 g/kg. These results are comparable with sesame oils, which are well-known for their high lignan content (approximately 11 g/kg) [23], indicating that the studied oil is a good source of lignans, which are known to possess a range of bioactivities, such as anti-inflammatory, anti-oxidative and immunomodulatory [38].

### 3.3. Nutritional Evaluation of CPE Oil

The oil of *C. cardunculus* cultivar, obtained through the CPE method—a green and safe method for consumers—was examined for its nutritional value (Table 2). The macronutrient analysis was performed by the determination of several parameters (energy, protein, fatty acids, ash, dietary fibers) concluding that oil obtained from CPE method contained higher energy content, even from the original seeds [20], as well as higher energy and protein content compared to sesame- and flax-seed oils [39,40].

### 3.4. Total Phenolic Content (TPC)

The TPC of the five oils was found notably different when performed ANOVA (*p* < 0.0001) assuming unequal variances of populations. Multiple comparison test (Dunnett’s approach, recommended for small sample size) showed significant difference, among SE vs. sesame (*p* < 0.0001), flaxseed (*p* = 0.0405) and olive oils (*p* = 0.0050), among CPE vs sesame (*p* = 0.0003), and olive (*p* = 0.0028), while no significant difference was observed for CPE vs. flaxseed (*p* = 0.0580). No significant difference was observed between CPE and SE, with a *p* value calculated at 0.2470. The observed difference of CPE and SPE with flaxseed can be expected from the marginal *p* values (0.0405 and 0.0580, respectively). It is noteworthy that the TPC of both *Cynara* cultivar oils was higher, compared with sesame, flaxseed and even olive oils (Table 3). These results are in accordance with literature data, in which oil obtained from *C. cardunculus* from Chile exhibited high phenolic content, comparable with that of edible olive oil of the same origin [8]. In our case, oils from the Greek cultivar of *C. cardunculus* showed TPC value at least double higher than the olive oil (selected to be one of the best qualities) and higher than the flaxseed and sesame oils, which are widely consumed, in our days.

The TPC value of the seedcake was also determined, exhibiting a higher phenolic content compared to the seeds [20] indicating that significant amounts of phenolics remain in the seedcake after CP oil extraction. These results are in accordance with previously reported data about seedcakes obtained from seeds of cultivated cardoon [9].

### 3.5. Antioxidant Activity

In order to assess the antioxidant properties of the SE and CPE oils, their free radical-scavenging activity was estimated by DPPH^•^ and ABTS^•+^ assays and compared with those of sesame, flaxseed and olive oils (Table 4 and Table 5). Moreover, for comparison reasons, the seedcake and isolated compounds from the seeds (arctigenin, trachelogenin, arctiin, tracheloside, cynarinine, 3,5-dicaffeoylquinic acid) that were also detected in the seedcake were evaluated.

According to the results and concerning the tested compounds, 3,5-dicaffeoylquinic acid exhibited the highest inhibition, even in the lower concentration of both assays (approximately 50% inhibition), while arctigenin and trachelogenin were found to be interesting antioxidant agents. According to reported data about the antioxidant potential of caffeoylquinic acids [41], 3,5-dicaffeoylquinic acid was the acid that demonstrated the highest activity, which is in accordance with the present findings. Arctigenin has been previously evaluated and compared with arctiin for its antioxidant properties [42], in which it was concluded that arctigenin possessed the higher antioxidant activity, while trachelogenin exhibited weaker activity than arctigenin. Furtehrmore; to the best of our knowledge, this is the first report on the antioxidant activity of cynarinine.

The seedcake, as a remaining “pool” of large amounts of phenolics after oil extraction from the seeds, exhibited high antioxidant activity and it could be suggested as a promising bioactive plant material, instead of a discarded by-product.

### 3.6. Enzyme Inhibitory Properties

AChE inhibitors are linked with cognitive enhancement, as well as with reduced risk for mortality, which could be attributed to their cognitive effects, and they may potentially see use in the treatment of Alzheimer’s dementia [43]. The determination of the enzyme inhibitory activity of the CPE oil and seedcake are presented in Table 6, showing that the seedcake exhibited better effects in comparison with the CPE oil.

The seedcake also showed a very high total phenolic content, which could be attributed to metabolites with caffeoyl- groups responsible for the observed AChE inhibitory activity that depends on phenolic compound’s structure, as well as the number and position of their hydroxyl groups [43]. Furthermore, lignans, such as arctigenin and arctiin, which were isolated from the seeds, have been previously assayed for their memory-enhancement effects, exhibiting promising results [44].

## 4. Conclusions

In this study, seed oils obtained by two different extraction methods (CPE and SE) from an established GCCC were analysed for the first time. The produced oils were compared with respected and highly consumed edible vegetable oils, such as sesame, flaxseed and olive oils. The seedcake—the by-product obtained after cold-press oil extraction—together with isolated phenolic secondary metabolites found in both seeds and seedcake, were also biologically evaluated for their antioxidant and anti-AChE activities.

In both studied oils, polyunsaturated fatty acids were predominant; linoleic acid was the abundant compound, followed by monounsaturated and saturated fatty acids. Moreover, high lignan content was determined in both oils, while an interesting nutrient profile of CPE oil, comparable with other respected edible oils, was recorded.

The present analysis indicates that the GCCC oil obtained through the CPE method—a low-cost, green and eco-friendly method for consumers, as it is free from enormous volumes of toxic organic solvents that classic extraction techniques requires—could be included in the list of functional foods as a potential candidate for properties of human health, with respect to its chemical and nutritional composition, together with its high phenolic and lignan content, as well as its revealed activity against acetylcholinesterase.

Moreover, seedcake, which represents approximately 80% of the mass fraction and used mainly as organic fertilizer and/or animal feed [12], also proved to be a rich source of phenolics, with interesting antiradical potential and AChE inhibitory activity connected with neuroprotective potential. Thus, it could be further exploited as a novel and valuable supplement in food and in the pharmaceutical industry.

Conclusively, since there is an increasing interest in products of natural origin with high bioactive properties, GCCC seeds appear to be an excellent plant source for the exploitation of added-value crude material and bioactive metabolites for utilization in the food industry, but also with promising future applications in the pharmaceutical and/or cosmetics industries.

## Figures and Tables

**Table 1:** Determination of fatty acid composition in a GCCC seed oil obtained by SE and CPE.

Fatty Acid	SE (%)	CPE (%)
Myristic acid	0.21 ± 0.08	0.31 ± 0.06
Palmitoleic acid	0.31 ± 0.02	0.30 ± 0.02
Palmitic acid	16.22 ± 0.10	20.42 ± 0.08
Margaric acid	0.08 ± 0.01	0.07 ± 0.03
Linoleic acid	57.23 ± 0.57	47.23 ± 0.40
Oleic acid	17.31 ± 0.10	28.02 ± 0.9
Stearic acid	3.71 ± 0.03	3.42 ± 0.3
Linolenic acid (γ-linolenic acid/α-linolenic acid)	0.47 ± 0.02	nd
Linolenic acid (γ-linolenic acid/α-linolenic acid)	0.74 ± 0.01	nd
Arachidic acid	0.34 ± 0.05	0.10 ± 0.04
Behenic acid	0.07 ± 0.04	nd
SFA	20.63 ± 0.25	24.32 ± 0.09
MUFA	17.62 ± 0.08	28.32 ± 0.88
PUFA	58.44 ± 0.37	47.23 ± 0.40
PUFA/SFA	2.83 ± 0.3	1.94 ± 0.02

C14:0, myristic acid; C16:0, palmitic acid; C17:0, heptadecanoic acid-margaric acid; C18:0, stearic acid; C20:0, arachidic acid; C16:1, palmitoleic acid; C18:1, ω-9, oleic acid; C18:2, ω-6, linoleic acid; C18:3, ω-6, γ-linolenic acid; C18:3, ω-3, α-linolenic acid; SFA, saturated fatty acids; MUFA, monounsaturated fatty acids; PUFA, polyunsaturated fatty acids. nd = not detected, Results were expressed as means ± standard deviation (*n* = 3).

**Table 2:** Nutritional aspects of CPE oil.

Physicochemical Parameters	Value
energy	892 Kcal/100 g 3666 Kj/100 g
moisture content	<0.1%
protein content	1.7 ± 0.4%
saturated fatty acids	16.1 ± 0.3%
unsaturated fatty acids	83.9 ± 1.2%
fat content	23.7 ± 0.5%
ash content	<0.1%
dietary fibers	<0.1%

Results were expressed as means ± standard deviation (*n* = 3).

**Table 3:** Determination of TPC in seed oils of GCCC (SE and CPE), three other vegetable oils and seedcake.

Oils	TPC (g GAE/kg Oil)
SE oil	4.7 ± 0.1
CPE oil	5.1 ± 0.1
Sesame oil	0.1 ± 0.2
Flaxseed oil	3.0 ± 0.4
Olive oil	2.1 ± 0.3
	**TPC (mg GAE/g extract)**
Seedcake	111.4 ± 1.7

Results were expressed as means ± standard deviation (*n* = 3).

**Table 4:** Determination of the antioxidant activity of the studied oils, seedcake and phenolics by DPPH^•^ assay.

DPPH^•^				
Tested Materials	% Inhibition 200 μg/mL	% Inhibition 100 μg/mL	% Inhibition 50 μg/mL	% Inhibition 25 μg/mL
SE oil	NA	ND	ND	ND
CPE oil	NA	ND	ND	ND
sesame oil	NA	ND	ND	ND
flaxseed oil	NA	ND	ND	ND
olive oil	NA	ND	ND	ND
Seedcake	78.94 ± 1.25	62.32 ± 0.55	40.36 ± 1.13	5.40 ± 0.75
arctigenin	69.96 ± 0.10	55.09 ± 0.17	38.83 ± 0.49	ND
trachelogenin	58.60 ± 0.36	43.01 ± 0.32	29.06 ± 0.20	ND
arctiin	31.72 ± 0.18	ND	ND	ND
tracheloside	NA	ND	ND	ND
cynarinine	29.03 ± 0.17	ND	ND	ND
3,5-dicaffeoylquinic acid	90.54 ± 0.06	90.25 ± 0.05	87.54 ± 0.10	50.90 ± 0.47

Results were expressed as means ± standard deviation (*n* = 3), NA: not active, ND: not detected.

**Table 5:** Determination of the antioxidant activity of the studied oils, seedcake and phenolics by ABTS^•+^ assay.

	ABTS^•+^									
Tested Materials	% Inhibition 200 μg/mL			% Inhibition 100 μg/mL				% Inhibition 50 μg/mL		
SE oil	2.97 ± 0.91			ND				ND		
CPE oil	6.37 ± 0.30			ND				ND		
sesame oil	1.08 ± 0.16			ND				ND		
flaxseed oil	2.38 ± 1.44			ND				ND		
olive oil	4.06 ± 0.66			ND				ND		
	% Inhibition 200 μg/mL		% Inhibition 100 μg/mL			% Inhibition 50 μg/mL			% Inhibition 25 μg/mL	
Seedcake	99.93 ± 0.11		98.02 ± 0.24			74.52 ± 0.12			48.86 ± 0.28	
	% Inhibition 200 μg/mL	% Inhibition 100 μg/mL			% Inhibition 50 μg/mL		% Inhibition 25 μg/mL			% Inhibition 12.5 μg/mL
arctigenin	99.68 ± 0.14	99.46 ± 0.13			97.03 ± 0.08		85.68 ± 0.75			52.34 ± 1.25
trachelogenin	99.33 ± 0.14	98.00 ± 0.10			93.62 ± 0.10		77.66 ± 0.30			48.07 ± 0.21
arctiin	93.05 ± 0.36	77.15 ± 0.60			53.63 ± 0.45		20.80 ± 0.55			4.14 ± 0.65
tracheloside	15.37 ± 0.57	0.91 ± 0.73			NA		NA			NA
cynarinine	94.38 ± 0.18	88.20 ± 0.28			77.29 ± 0.41		64.99 ± 0.22			37.69 ± 0.73
3,5-dicaffeoylquinic acid	100.28 ± 0.14	100.58 ± 0.14			100.73 ± 0.11		100.77 ± 0.24			53.64 ± 0.61

Results expressed as means ± standard deviation (*n* = 3), NA: not active, ND: not detected.

**Table 6:** Determination of the enzyme inhibitory activity of CPE oil and seedcake.

	AChE Inhibition Assay		
	% Inhibition 200 μg/mL	% Inhibition 100 μg/mL	% Inhibition 50 μg/mL
CPE oil	22.79 ± 4.25	4.76 ± 3.10	8.15 ± 2.21
seedcake	32.44 ± 2.46	18.64 ± 7.67	0.27 ± 4.42

Results expressed as means ± standard deviation (*n* = 3).

## Data Availability

Not applicable.
